# Hereditary gynaecologic cancers in Nepal: a proposed model of care to serve high risk populations in developing countries

**DOI:** 10.1186/s13053-017-0072-y

**Published:** 2017-09-18

**Authors:** Hanoon P. Pokharel, Neville F. Hacker, Lesley Andrews

**Affiliations:** 1 Gynaecologic Cancer Centre, Royal Hospital for Women, Sydney, Australia; 2 School of Women’s and Children’s Health, UNSW, Sydney, Australia; 3 Hereditary Cancer Clinic, Prince of Wales Hospital, Sydney, Australia; 4 Department of Obstetrics and Gynaecology, B.P Koirala Institute of Health Sciences, Dharan, Nepal; 5 School of Medicine, UNSW, Sydney, Australia

**Keywords:** Nepal, Hereditary cancer, BRCA, Lynch syndrome

## Abstract

**Background:**

Endometrial, ovarian and breast cancers are paradigms for global health disparity. Women living in the developing world continue to present in later stages of disease and have fewer options for treatment than those in developed countries. Risk reducing surgery is of proven benefit for women at high risk of gynaecological cancer. There is no specific model for identification and management of such women in the developing world.

**Methods:**

We have integrated data from our published audit of a major gynaecological oncology centre at Royal Hospital for Women in Australia, with data from our survey and a focus group discussion of Nepalese gynaecological health care professionals regarding genetic testing, and findings from the literature. These data have been used to identify current barriers to multidisciplinary gynaecological oncology care in developing nations, and to develop a model to integrate hereditary cancer services into cancer care in Nepal, as a paradigm for other developing nations.

**Results:**

The ability to identify women with hereditary gynaecological cancer in developing nations is influenced by their late presentation (if active management is declined or not appropriate), limited access to specialised services and cultural and financial barriers. In order to include genetic assessment in multidisciplinary gynaecological cancer care, education needs to be provided to all levels of health care providers to enable reporting of family history, and appropriate ordering of investigations. Training of genetic counsellors is needed to assist in the interpretation of results and extending care to unaffected at-risk relatives. Novel approaches will be required to overcome geographic and financial barriers, including mainstreaming of genetic testing, telephone counselling, use of mouth swabs and utilisation of international laboratories.

**Conclusion:**

Women in Nepal have yet to receive benefits from the advances in early cancer diagnosis and management. There is a potential of extending the benefits of hereditary cancer diagnosis in Nepal due to the rapid fall in the cost of genetic testing and the ability to collect DNA from a buccal swab through appropriate training of the gynaecological carers.

## Background

Poor health within countries and inequities between countries are largely caused by the unequal distribution of power, income, goods and services, resulting from a combination of poor social policies, unfair economic arrangements and bad politics [1]. Cancer has historically been given low priority in developing countries, particularly from bilateral and multilateral aid agencies, but also domestically. Only 5% of global spending on cancer is directed toward developing countries, which have 80% of the world’s population [2]. By 2020, the International Agency for Research on Cancer, a branch of the World Health Organization (WHO), predicts 16 million new cases of cancer per year, with cancer overtaking heart disease to become the world’s number 1 killer. Currently 12.5% of all deaths are caused by cancer, which is more than HIV/acquired immunodeficiency syndrome (AIDS), tuberculosis, and malaria combined [3].

Advances in health information technology, and the emergence of new models of health care financing that support innovation in the delivery of preventive care, have created an important opportunity to develop a novel and sustainable approach to address these challenges [4].

Nepal is a small landlocked country, between the world’s two largest growing economies, India and China. The population of approximately 30 million has an estimated 50,000–70,000 new cases of cancer diagnosed per year [5].

In 2014, the leading causes of cancer mortality in women in Nepal were cervical (18.4%), breast (11.6%), endometrial (9.5%) and ovarian cancer (7%) [6]. This contrasts with female cancer mortality in Australia, where the leading causes of cancer mortality are lung (17.0% of cancer deaths), breast (14.5%), colorectal (9.4%) and pancreatic cancer (6.4%). Cervical cancer accounts for only 1.1%, uterine 2.5% and ovarian cancer 5% of female cancer deaths [7]**.** These differences reflect lower rates of lifestyle related cancers in Nepal, as well as higher mortality from cancers which are preventable or curable in developed nations.

Despite a significant cancer incidence and mortality, Nepal has yet to establish an operational cancer policy, strategy or action plan. A cancer registry supported by the World Health Organisation (WHO) was established in 2005 to collect cancer data from seven major hospitals; however data from much of the country’s population remains uncollected [8].

Geography imposes severe restrictions on the provision of health services in many low income countries where challenges exist in reaching populations in remote, mountainous locations [9–11]. In Nepal for example, the utilisation of a health facility for the delivery of a child is considerably lower in the mountains than in the country’s two other ecological regions, the terai (i.e. the plains) and the hills. Coverage of health services in the mountains is approximately half the percentage observed in the terai and less than two-thirds the percentage reported in the hills [12]. Geographical inaccessibility to health services features in the mountains owing to scarce and dispersed populations, tough terrain with no transport or difficult road conditions, seasonal isolation, and remoteness [13]. Geographical barriers to health care are a common feature of developing nations and contribute to the low uptake of preventive strategies and the high cancer mortality.

### Hereditary gynaecologic cancer

A gynaecological cancer diagnosis may identify women at risk of germline mutations in BRCA1, BRCA2 or in the mismatch repair (MMR) genes**.** Since the identification of the BRCA1/2 cancer susceptibility genes in 1994 [14, 15], there have been significant advances in our knowledge of the cancer risks for mutation carriers. Meta-analyses indicate that a BRCA1 mutation carries a 47–66% probability of developing breast cancer and a 35–46% probability of developing ovarian cancer over a woman’s lifetime and with a BRCA2 mutation, it is 40–57% for breast and 13–23% for ovarian cancer [16].There is wide differences in BRCA1/2 mutation penetrance estimates between individual studies. This variation may be due to ascertainment differences, or reflect racial or ethnic differences in genetic, reproductive, or environmental exposures which could have significant implications for clinical practice, particularly for endogamous populations in developing nations [17–19]**.**

Lynch syndrome, also referred to as Hereditary Non-Polyposis Colorectal Cancer (HNPCC), is an autosomal dominant disease that may lead to colorectal, endometrial, gastric and ovarian cancer. Lynch syndrome is caused by germline mutations in DNA mismatch repair (MMR) genes, which include MLH1, MSH2, MSH6 and PMS2. The estimated risk of developing endometrial, colorectal and ovarian cancer for patients with MMR gene mutations by the age of 70 years is 15–30%, 25–37% and 8–15% respectively [20–23]. There are considerable variations between risks associated with each gene. Protocols exist for performing mismatch repair expression immunohistochemistry on endometrial and ovarian cancer to screen for evidence of Lynch Syndrome [24–26]. There is current debate as to whether all endometrial cancers should undergo MMR immunohistochemistry, irrespective of age at diagnosis, or only those diagnosed under age 60 or 70. However, as methylation of MLH1 as a cause of deficient expression becomes more common at older ages, there is a greater need for reflex methylation studies without an age limit for immunohistochemistry. This needs to be balanced against the potential to miss women with Lynch Syndrome diagnosed at older ages.

Compared to sporadic endometrial cancer, the characteristics of endometrial cancer in Lynch syndrome include trend towards a lower rate of endometrioid tumours (86.0 vs. 97.6%), but conversely a higher incidence of patients with FIGO stage I-II disease (88.0 vs. 73.8%) [27]; ovarian cancers are more commonly epithelial tumours (95.9 vs. 83.6%), including endometrioid (18.3 vs. 9.6%) and clear cell tumours (18.3 vs. 3.6%) [28].

Timely genetic testing of selected patients may influence treatment, enable concurrent or future risk-reducing screening and surgery, and provide opportunities for identification of at-risk relatives [29]. Multiple economic evaluations taking into consideration geographical, ethnic and temporal variations have shown that genetic testing to identify unaffected hereditary cancer mutation carriers is a cost effective intervention if followed by appropriate screening and/or risk reducing interventions. For unaffected female BRCA1/2 mutation carriers, an extensive cost-effectiveness study found that prophylactic oophorectomy along with bilateral prophylactic mastectomy was cost-effective [$US2352 per life-year saved (LYS)] when compared with a standard United States cost-effectiveness threshold of $50000–100,000 per LYS [30]. Risk reducing salpingo-oophorectomy is a minimally invasive procedure which not only dramatically reduces ovarian and fallopian cancer risk, but also reduces the risk of breast cancer, particularly if done pre-menopausally. It has been shown to be associated with a lower risk of ovarian cancer, a lower risk of breast cancer, lower all-cause mortality, breast cancer-specific mortality, and ovarian cancer-specific mortality in BRCA1/2 mutation carriers. The most effective single intervention for BRCA1 mutation carriers is salpingo-oophorectomy at age 40, yielding a 15% absolute survival gain [31, 32].

Prophylactic hysterectomy with bilateral salpingo-oophorectomy is an effective strategy for preventing endometrial and ovarian cancer in women with Lynch syndrome [33]. Annual colonoscopy has proven benefits in reducing cancer incidence in mis-match repair mutation carriers [34]. In resource poor developing nations, these screening and risk-reducing interventions have the potential to reduce the cancer burden in a cost-effective manner.

While guidelines exist for the identification, investigation and management of families with hereditary gynaecological cancer in developed countries including Australia, the USA and UK, we did not find any evidence in the published literature regarding protocols for hereditary gynaecological cancer identification in developing nations such as Nepal. This paper seeks to describe the essential processes of BRCA1/2 and mismatch repair mutation carrier identification following a diagnosis of gynaecological cancer, as demonstrated at the Royal Hospital for Women, Sydney, and identify the barriers to, and requirements for, implementing such a protocol in Nepal, as an example of a developing nation.

## Methods

Initially, we reviewed the management of patients at a major gynaecological oncology centre in Sydney, Australia, with regard to identification of potential carriers of inherited mutations in BRCA1, BRCA2 or the mismatch repair genes [29]. We have used this protocol as a model of care in a developed nation.

The second arm involved gathering information from Nepalese gynaecologists and other health care professionals regarding their experience and attitudes to genetic testing [35]. Further, we conducted a focus group discussion among the senior gynaecologists practising in Nepal for more than a decade. The discussion was conducted to find out the possibility of starting hereditary gynaecologic oncology services and initiating a hereditary gynaecologic oncology registry in Nepal.

We have integrated these data with available literature for the third arm of our study: an analysis of the management of gynaecological cancer in Nepal; identification of barriers to multidisciplinary care including hereditary gynaecological cancer assessment, and development of a model to integrate hereditary cancer services into cancer care in Nepal, as a paradigm for other developing nations.

## Results

### Protocol for identification of women at risk of hereditary gynaecological cancer in a developed nation

**Fig. 1:**
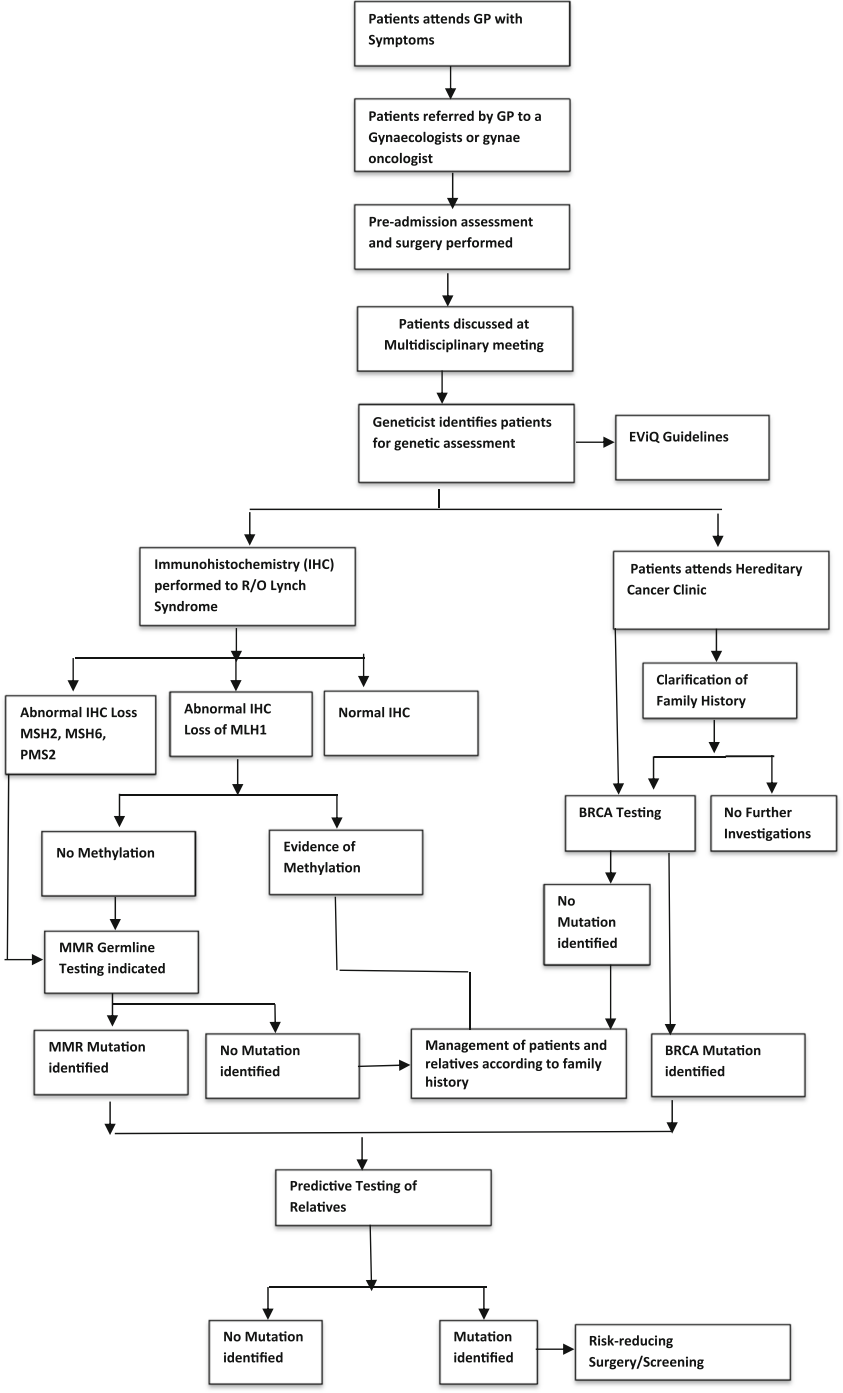
Algorithm for the identification of patients requiring referral for hereditary assessment at the Gynaecologic Cancer Centre at the Royal Hospital for Women in Sydney

The Royal Hospital for Women (RHW) in Sydney, Australia has a specialised gynaecological oncology service. Following diagnosis, all cases of gynaecological cancer are reviewed at a multidisciplinary meeting. A member of the genetics team is in attendance, and identifies those women suitable for further genetics assessment. This may include clarification of family history, Mismatch Repair Immunohistochemistry (MMR IHC), methylation studies and/or genetic testing. A typical patient course is represented in Fig. 1.

During the 5 year period of our previously reported audit [29], The Royal Hospital for Women, Sydney treated 2523 women for gynaecological cancer (average 500 per year). Of those, 462 women were recommended for referral to the hereditary cancer clinic. Pathogenic mutations were identified in 40 of 165 women (24%) undergoing testing for BRCA1/2 genes and in 10 of 25 women (40%) who underwent MMR genetic testing. Eighty- seven first or second degree relatives of those women underwent predictive testing, which identified 47 mutation carriers and 40 non-carriers.

### The existing cancer care process in Nepal

In Nepal, the patient attends the family medicine outpatient department and is then referred to the specialized gynaecological outpatient department. In the outpatient department, the doctors take a detailed history. This includes the patient’s name, age, address, educational level and the name of the nearest relative. This is likely to be the individual who will most likely be making important decisions in the family.

Appropriate clinical history, physical examination and radiological or medical investigations are performed. If gynaecological cancer is diagnosed or suspected, counselling is initially done with the relative, which is considered appropriate to Nepali culture [36].

If the disease is considered operable, appropriate surgery is undertaken in the tertiary care hospitals, or the patient is referred to a specialized cancer centre such as B P Koirala Memorial Cancer hospital in Bharatpur or Bhaktapur Cancer Hospital in Kathmandu. If the disease is not considered operable, a histological or cytological diagnosis is obtained, and the necessary investigations performed to allow FIGO staging.

### Barriers to implementation of a hereditary cancer identification process in a developing nation

Although there have been recent advances in the availability of genetic testing in many developing countries, there is disparity in skills and knowledge among the health care providers regarding the availability of modern scientific tools, and recognition of the benefits of genetic testing for gynaecological cancers. As such, there is a great need for the introduction of further genetic counselling education and hereditary cancer services for gynaecological care in Nepal [35]. However, multiple barriers need to be overcome.

#### Late presentation

Limited public awareness of the symptoms of gynaecological cancers is a major barrier for the early diagnosis of gynaecological cancers in developing nations such as Nepal. For example, a woman who has attained menopause may not know that post-menopausal bleeding may be a presenting symptom for many gynaecological cancers, which otherwise could be cured if diagnosed in the early stages.

There is also a lack of awareness about the prognosis of gynaecological cancer, as most patients, family members, and many health care professionals consider cancer to be an incurable disease. This leads to delay in presentation of patients to hospital, thus increasing the number of advanced stage cancers, negatively impacting on rates of morbidity and mortality and reinforcing the belief that cancer is incurable. Many patients therefore are never seen by a specialised service.

#### Limited access to specialised services

There is mal-distribution of health services, with specialised health services being available only in the two major cities of the country. There is no access to specialised health care services or diagnostic facilities in other parts of the country. There are only four centres in the country treating cancer, three of which are located within greater Kathmandu. [5].

Expertise in the multidisciplinary management of gynaecological cancers in Nepal is limited. Currently there are no qualified genetic counsellors or cancer geneticists in Nepal with counselling generally performed by the gynaecologists. There are also no laboratories performing routine tumour IHC or genetic testing for hereditary cancer.

#### Low health literacy

With a health literacy rate of 57% and 66% in females and males respectively [37], many Nepalese patients may not have the capacity to obtain and understand the basic health information necessary to be able to make appropriate health decisions. One of the biggest challenges to improving health outcomes is educating women and empowering them with the health knowledge necessary to seek appropriate health care and make informed decisions. This is particularly relevant with regard to hereditary cancers, as women in developing nations are unlikely to be well informed about opportunities for genetic testing and preventive and risk reducing strategies.

Family history is a key component of identifying patients at risk of hereditary cancer. Poor health literacy impairs the reporting of personal and family cancer history, and is a major barrier to accurate genetic assessment. There is a need for greater awareness of the significance of family history in cancer risk prediction amongst both primary care providers and patients.

Further to this, the outflow of patients going to neighbouring India for treatment results in under-reporting of cancer cases by family members, and the absence of a standardized central registry further impedes the clinician’s ability to obtain an accurate family history.

#### Cultural, religious and traditional health practices

Persisting secrecy around cancer diagnosis inhibits knowledge of family history, and is a major barrier to the recognition of hereditary cancer syndromes. Traditionally, doctors in Nepal do not discuss a diagnosis of malignancy with their patients. Instead, discussion is more likely to take place with family members, who then filter the information that the patient receives. This is accepted by the professionals in Nepal as an unchallengeable tenet of Nepal’s illness culture [36]. Women diagnosed with cancer may not be in a position to make autonomous decisions about genetic assessment. The complexity of family dynamics may inhibit the treating doctor’s willingness to introduce hereditary factors into the patient’s management. Additionally, passing on information to patients in resource-poor countries, may be limited if the health professional assesses that the patient is unable to afford any potential treatment options [38]. This may be particularly true of referral for genetic testing, where the benefits may not be perceived to take priority over other financial burdens.

Cancers that affect primarily women are a special subset of health inequity, as many women in developing countries lack access to screening and treatment as a consequences of discriminatory beliefs and practices [39]. This bias may adversely affect both clinician and family attitudes to genetic testing and risk management.

#### Geographical barriers

The topography of Nepal impedes access to medical services, impacting most on preventive health care and asymptomatic screening [40]. In developing countries such as Nepal, testing for hereditary cancers may be impaired by inadequate infrastructure for the transportation of samples (particularly from remote areas) to high-quality laboratories for processing and interpretation. Additionally, the lack of a computerized database to facilitate the follow-up of identified mutation carriers may limit the provision of predictive testing of relatives and implementation of risk reducing strategies.

#### Economic barriers

**Fig. 2:**
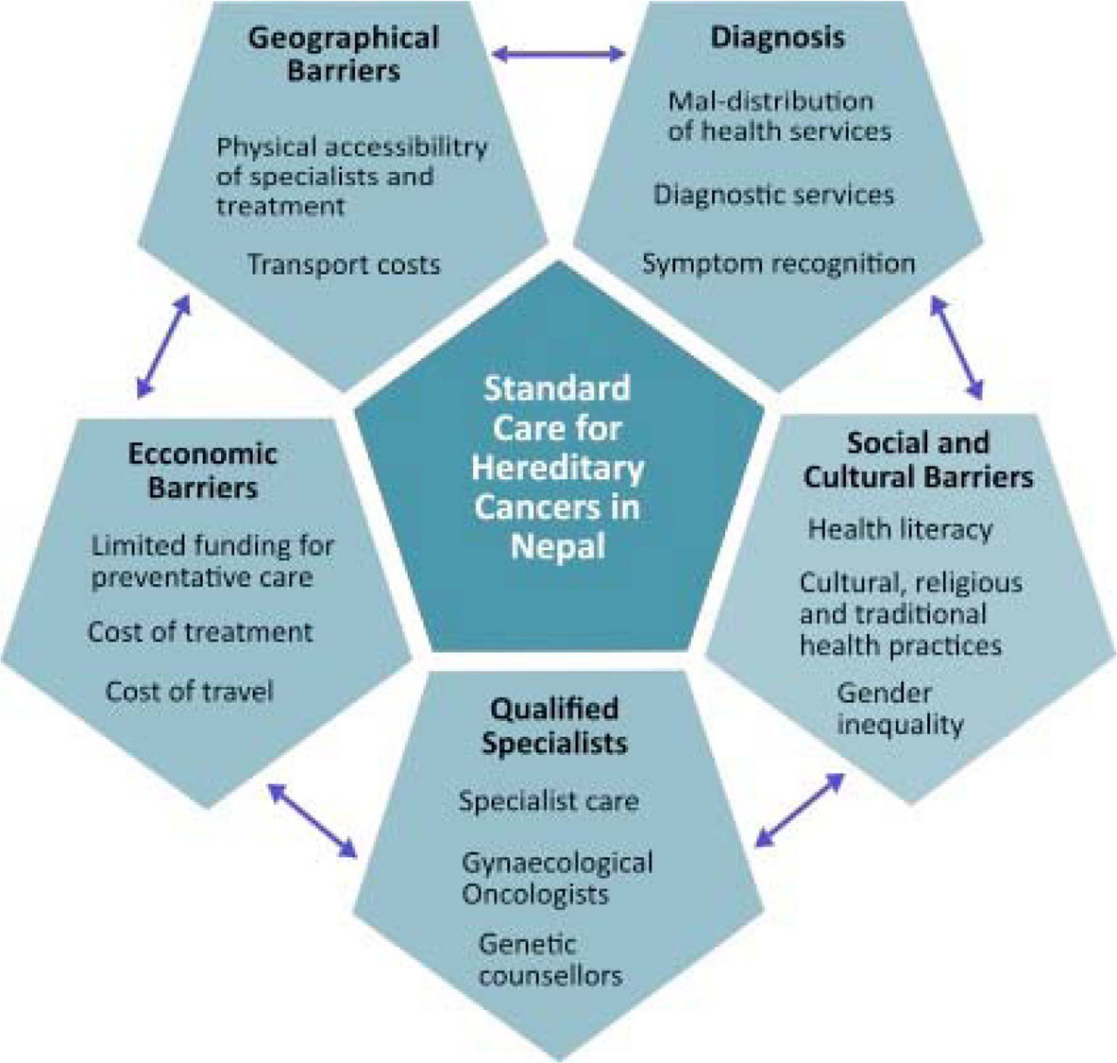
Barrier to establishing standard care for hereditary gynaecologic cancer patients in Nepal

Diagnosed patients may not seek treatment because of financial barriers prohibiting many families from receiving potentially lifesaving treatment. With a per capita income of US$600, many Nepalese are unable to afford expensive cancer treatments due to the lack of publicly funded health care or proper health insurance. The indirect cost in the treatment process is often overlooked (transport, accommodation and meals). Despite the gradually reducing trend in cervical cancer diagnosis as a result of the Human Papillomavirus (HPV) vaccine in developed nations, cervical cancer remains a priority in developing countries. Along with this, hereditary cancers face significant competition with infectious diseases, maternal mortality and other health needs vying for the limited resources, infrastructures, laboratory facilities and scarcity of qualified health professionals (Fig. 2).

## Discussion

### A model for developing nations

#### Estimating the burden of disease

BRCA1 and 2 account for 14.1% of non-mucinous ovarian cancers, 17% of serous cancers and 3% of endometrial cancers in Australia [26, 41]. These cancers are potentially preventable with the identification of mutation carriers and timely risk-reducing surgery. However, until genetic testing becomes widespread in developing nations, the prevalence of mutations, and hence the burden of hereditary gynaecological cancer in individual countries will remain unknown. Similarly, the mutation spectrum is unknown. It is likely that in endogamous, geographically isolated communities, a small number of founder mutations may account for the majority of hereditary gynaecological cancer. Once the mutation prevalence and spectrum is known, testing may be able to be restricted to a small number of mutations, decreasing the cost considerably, as in the Ashkenazi Jewish community, which has a high prevalence of 3 specific founder mutations**.**

A large majority of gynaecological cancers in Nepal are treated at the B.P. Koirala Memorial Cancer Hospital (BPKMCH). Assuming a similar mutation prevalence, penetrance and uptake of testing to Australia, the introduction of a protocol at BPKMCH similar to that at the RHW in Sydney, would identify 94 BRCA1/2 mutation carriers and 23 MMR gene mutation carriers annually through the BPMCH and enable predictive testing of a further 204 relatives. Most importantly, it would enable a further 110 identified unaffected mutation carriers to be advised of risk management strategies (Table 1).

**Table 1:** Extrapolated burden of hereditary gynaecologic cancers at one major gynaecologic cancer centre in Nepal

	RHW	BPKMCH
Total number of patients admitted	2523 (2010–2014- audit)	4901 (2014-annual report)
Patients recommended for referral(5 years) to HCC	462	–
Total number of patients of ca ovary and endometrium/year	345	809
Mutation Identified for BRCA1/2	40	*94 Extrapolated*
Mutation Identified for MMR	10	*23*
Predictive testing	87	*204*
Mutation Carriers	47	*110*

RHW Royal Hospital for Women, BPKMCH B.P. Koirala Memorial Cancer Hospital

#### Guidelines for gynaecologists

While BRCA1/2 mutations are estimated to be carried by 1 in 500 individuals in a western population, the prevalence in other populations such as Nepal is unknown. Additionally, the existence of population specific founder mutations will remain unknown until genetic testing becomes more common. Emerging data on mutations across race/ethnicity will influence how best to target clinical testing. In developed countries, mutation risk assessment is performed by genetic counsellors, using predictive models that improve on clinical expertise. In hereditary breast ovarian cancer, the most commonly used predictive models are BRCAPRO, Myriad II and BOADICEA [42–44]. Recent studies however have reported there is up to six-fold underestimation of Asian mutation carriers with BRCAPRO and Myriad II models [45, 46] potentially limiting their use in developing countries. Further studies are required to determine the most effective model to use with specific populations. However, until such data are available, guidelines used in developed nations such as Australia (https://www.eviq.org.au) should be adopted to estimate if a woman has at least a 10% chance of carrying a mutation, which is the commonly adopted threshold for testing.

#### Development of educational module screen for care providers

In developing countries, in order to make an impact in the management of gynaecological cancer, the treating gynaecologist should be trained and skilled in counselling techniques which will ultimately maximise the detection of mutation carriers. The information applies to a relatively small number of patients in any practice, and the evidence base changes rapidly, making it difficult for all but the most specialized providers to stay up-to date. In our previous study [35] it was found that approximately 46% of all clinicians felt prepared to answer patient’s questions about genetic testing for gynaecologic cancer. However, while 94% of gynaecologic oncologists felt prepared, only 47% of gynaecologists and 26% of other clinicians did so, despite 86% of gynaecologists and 71% of other clinicians reporting that patients had asked questions about genetic testing [35]. An educational program could be devised, based on the emerging practice of “mainstreaming”, i.e. handing over testing of affected women to gynaecologists who have undergone a short training module. This would include key aspects of providing culturally appropriate informed consent prior to germline testing Specialised genetic services would only be consulted if a mutation was identified [47]. One of the benefits of mainstreaming is that patients do not need referral to a separate service for genetic testing. According to a previous audit at RHW prior to the introduction of mainstreaming, one third of referrals did not attend a hereditary cancer service for assessment/genetic testing. We have demonstrated that a systematic approach by the gynaecologist is required including short term follow-up (3 months) as longer term follow up may result in the loss of up to 36% of referred patients if an external referral is required [29]. Testing directly by the gynaecologist would minimise loss of patients who do not follow up their referral to a genetic clinic.

#### Improved health literacy

Great improvements in patient outcomes can be achieved by focusing on cooperation, health literacy and women’s rights. The patient’s ability to make decisions in favour of genetic assessment may improve their health outcome. Communicating this to affected family members also results in interventions with favourable health outcomes. In order to improve acceptance of genetic testing and empower women in developing nations to be more proactive in their health care, a simple document explaining hereditary cancer and the benefits of testing, especially for unaffected relatives, could be introduced at all women’s health clinics. The document would need to be presented in predominant local languages.

#### Testing processes

With decreasing costs of genetic testing, uptake of telephone counselling and use of readily transportable DNA collected through saliva or mouth swabs, we propose that distance should not be a barrier to this model being extended to all centres in Nepal which provide care to gynaecological cancer patients. Collection of human genomic DNA from buccal cells for genetic studies is readily accessible with a number of different methods available including cytobrush, mouthwash and treated cards. However studies show that cytobrush is preferred, because of its self-collection potential with good quality and sufficient quantity of DNA [48]. This method could be applied in Nepal’s setting, with samples easily transported to laboratories in India, or any other country until a cancer genetic laboratory is well established in Nepal. The same would apply to other developing nations. Molecular genetics services are gradually being introduced into Nepal, but at this time, testing for hereditary cancer predisposition genes is not available.

#### Predictive testing- access to genetic counsellors

In developed nations, predictive testing for hereditary cancer involves a genetic counsellor to aid the person in their understanding and decision making. The skills of the genetic counsellor are fundamental for the unaffected woman to understand the pros and cons of predictive testing and to ensure that any surgical interventions are made with fully informed consent. The profession of genetic counselling is virtually unknown in Nepal and many other developing nations. Until local universities incorporate this into their curriculum, genetic counsellors could be trained overseas, possibly with the provision of foreign financial aid.

#### Management of Unaffected Female Mutation Carriers

The greatest benefit of diagnosis of mutation carriers is the opportunity to identify unaffected female carriers so they may be offered screening and risk reducing options. While screening for ovarian cancer is not effective in the general population [49], some experts recommend that women with mutations in BRCA1 and 2, as well as women with strong family histories of breast or ovarian cancer have some form of screening with CA125, and transvaginal ultrasound, as often as every 3 months [50]. However, this schedule can cause considerable patient anxiety, and has not been shown to improve survival from ovarian cancer. To date, no macroscopic detectable precursor to ovarian cancer has been identified. Thus, women at increased risk are encouraged to undergo prophylactic removal of the ovaries and fallopian tubes around the age of 40 when they have completed childbearing, followed by hormone replacement until the age of 50. In the hands of trained gynaecologists, this can be achieved as a day only procedure with minimal operative risk. Once educated about the indications for this intervention, little further education of gynaecologists about the procedure would be required.

Women with Lynch Syndrome can be offered a hysterectomy and bilateral salpingo-oophorectomy with hormone replacement – core skills of existing gynaecologists in developing nations requiring no further education.

#### Financial support

In Nepal and other developing nations, the priorities, policies and protocols are more focused on treating the patients who are already diagnosed with cancer and are in need of treating physicians. Detection of hereditary cancer needs active interventions, but policy makers may not agree to fund this because of competing priorities.

Low- and middle income countries (LMCs) are urged to support research translating knowledge into effective public health measures for cancer prevention, to improve access to appropriate technologies for the early diagnosis and treatment of cancer, and to promote research evaluating low-cost interventions that are affordable and sustainable [51]. Although most LMCs have not yet identified cancer as a priority healthcare issue, in future it will become an important public health problem as the control of communicable diseases improves [52].

Developed nations have demonstrated that it is cost-effective to include identification of mutation carriers as standard of care. To address financial barriers there is a strong need for advocacy for an effective health insurance system or publicly funded hospitals, which should be adequately equipped with the facilities and expertise to incorporate hereditary cancer into standard care of the woman with gynaecological cancer. The services could be piloted through a public owned academic medical institution where the need for academic growth, availability of research facilities and the continuity of services can be ensured.

Despite a variety of impediments to health care service delivery and utilisation in Nepal, the need for specialised services such as hereditary cancer care cannot be ignored. Realistically, countries like Nepal are likely to be successful in developing such services only with support from cancer centres or academic groups from wealthier countries. Toward such a program, the RHW, UNSW, Sydney has committed to assisting by training doctors in advanced gynaecological cancer management through a memorandum of understanding. One aspect of this collaboration is the intention to transfer skills and knowledge necessary to establish a sustainable program of hereditary gynaecologic oncology services in Nepal. This provides a model for other partnerships.

## Conclusion

Although the position of women has improved substantially in Nepal and other developing nations over the past decades, progress has been uneven and multiple challenges remain. Despite great improvements in health in the past 30 years, many women are yet to benefit from these advances. Women’s low literacy levels, cultural and religious factors, competing health needs, discriminatory feeding patterns, limited resources, poorly developed health care services, and limited information on cancer prevention are contributory. However, with the rapidly falling cost of genetic testing, the ability to provide DNA from a stable buccal swab, and with appropriate training of gynaecological health carers, the model described here can be extended to other cancers associated with genetic predisposition, such as breast, colorectal and rare cancers to provide the benefits of hereditary cancer diagnosis to developing nations.
